# Signal transducer and activator of transcription STAT5 is recruited to *c*-*Myc* super-enhancer

**DOI:** 10.1186/s12867-016-0063-y

**Published:** 2016-04-14

**Authors:** Sophia Pinz, Samy Unser, Anne Rascle

**Affiliations:** Stat5 Signaling Research Group, Institute of Immunology, University of Regensburg, 93053 Regensburg, Germany

**Keywords:** STAT5, *c*-*Myc*, BET, BRD2, Super-enhancer, Chromatin

## Abstract

**Background:**

*c*-*Myc* has been proposed as a putative target gene of signal transducer and activator of transcription 5 (STAT5). No functional STAT5 binding site has been identified so far within the *c*-*Myc* gene locus, therefore a direct transcriptional regulation by STAT5 remains uncertain. *c*-*Myc* super-enhancer, located 1.7 Mb downstream of the *c*-*Myc* gene locus, was recently reported as essential for the regulation of *c*-*Myc* gene expression by hematopoietic transcription factors and bromodomain and extra-terminal (BET) proteins and for leukemia maintenance. *c*-*Myc* super-enhancer is composed of five regulatory regions (E1–E5) which recruit transcription and chromatin-associated factors, mediating chromatin looping and interaction with the *c*-*Myc* promoter.

**Results:**

We now show that STAT5 strongly binds to *c*-*Myc* super-enhancer regions E3 and E4, both in normal and transformed Ba/F3 cells. We also found that the BET protein bromodomain-containing protein 2 (BRD2), a co-factor of STAT5, co-localizes with STAT5 at E3/E4 in Ba/F3 cells transformed by the constitutively active STAT5-1*6 mutant, but not in non-transformed Ba/F3 cells. BRD2 binding at E3/E4 coincides with *c*-*Myc* transcriptional activation and is lost upon treatment with deacetylase and BET inhibitors, both of which inhibit STAT5 transcriptional activity and *c*-*Myc* gene expression.

**Conclusions:**

Our data suggest that constitutive STAT5 binding to *c*-*Myc* super-enhancer might contribute to BRD2 maintenance and thus allow sustained expression of *c*-*Myc* in Ba/F3 cells transformed by STAT5-1*6.

**Electronic supplementary material:**

The online version of this article (doi:10.1186/s12867-016-0063-y) contains supplementary material, which is available to authorized users.

## Background

*c*-*Myc* is a master regulator of essential biological processes such as cell proliferation, survival, differentiation, metabolism, angiogenesis and pluripotency establishment and maintenance [1, 2]. *c*-*Myc* is found overexpressed in most human cancers and is a hallmark of tumor initiation and maintenance [3, 4]. Characterizing the regulatory mechanisms of *c*-*Myc* gene expression is therefore fundamental for a better understanding of its deregulation in cancer and to possibly identify novel therapies aiming at controlling its expression. *c*-*Myc* gene transcription is regulated by multiple transcription factors via responsive elements located within both its promoter and remote enhancer regions [1, 5–9]. A number of reports, including those from our lab, provided evidence that *c*-*Myc* expression is regulated by signal transducer and activator of transcription 5 (STAT5) [10–13]. STAT5 is an essential regulator of cell differentiation, proliferation and survival [11, 14, 15] and is frequently constitutively activated in cancer. STAT5 constitutive activation results in *c*-*Myc* overexpression, increased cell proliferation and reduced cell apoptosis, and is as such an important player in cancer initiation and progression [11, 16–20]. Among molecules that suppress STAT5 activity, inhibitors of tyrosine kinases, of deacetylases and of bromodomain and extra-terminal (BET) proteins represent promising therapeutic agents, either alone or in combination [21–28]. We and others showed that expression of STAT5 target genes, including *c*-*Myc*, is reduced upon treatment with deacetylase inhibitors (trichostatin A, suberoylanilide hydroxamic acid, apicidin, valproic acid, sodium butyrate) or with the BET inhibitor (+)-JQ1 [12, 29–31]. Further elucidating the process of STAT5-induced expression of *c*-*Myc* and of its inhibition by clinically relevant therapeutic agents is critical for improving cancer therapy.

Among the evidence of a direct regulation of *c*-*Myc* by STAT5, an elegant study by Lord et al. demonstrated that *c*-*Myc* expression in response to IL-2 and IL-3 is dependent on the transactivation domain of STAT5 [10]. Accordingly, *c*-*Myc* expression is upregulated in cells expressing constitutively active STAT5, including in the BCR-ABL-transformed human leukemic cell line K562 [11–13, 32]. Constitutive activation of STAT5 by the oncogenic tyrosine kinase BCR-ABL contributes to K562 cell transformation [33, 34]. Overexpression of *c*-*Myc* in K562 cells is inhibited by the BCR-ABL tyrosine kinase inhibitor imatinib but also by the deacetylase inhibitor trichostatin A (TSA), which we showed to inhibit STAT5-mediated transcription [12, 31, 32]. In the same line, overexpression of *c*-*Myc* in Ba/F3 cells expressing the constitutively active STAT5 mutant 1*6 [35] is repressed by TSA [12, 31]. In apparent contradiction with a direct regulation a *c*-*Myc* by STAT5, we found that STAT5 knock-down in Ba/F3 cells did not affect IL-3-induced *c*-*Myc* gene expression [13]. Two other acknowledged direct target genes of STAT5, *Bcl*-*x* and *Id*-*1* [10, 13, 36–38], remained equally unaffected upon STAT5 knock-down in Ba/F3 cells [13]. Although these observations might be interpreted as an indication that these genes are not regulated by STAT5, they might also reflect an unconventional mechanism of regulation by STAT5, possibly not as sensitive to the partial (60 %) knock-down generated upon siRNA transfection [13]. Interestingly, in contrast to most classical STAT5 target genes (e.g., *Cis*, *Osm*, *Spi2.1*, …) which display STAT5 responsive elements within their promoter region [13], functional STAT5 binding sites have been identified outside the promoter regions of *Bcl*-*x* and *Id*-*1*, notably within *Bcl*-*x* first intron [36, 38] and within *Id*-*1* enhancer located several kb downstream of the *Id*-*1* gene [37]. These observations raise the possibility that regulation of *c*-*Myc* expression by STAT5 might be likewise unconventional, possibly involving distal elements. In support of this proposition, we previously attempted and failed to detect STAT5 binding along the *c*-*Myc* gene [13], including at GAS elements present in its promoter and known to mediate transcriptional response to other STAT family members [39–41].

Recently, several research groups described the role of a 3′ super-enhancer in the regulation of *c*-*Myc* gene expression in hematopoietic cells and its importance in *c*-*Myc* overexpression in leukemic cells [5–8, 42]. *c*-*Myc* super-enhancer is located 1.7 Mb downstream of the *c*-*Myc* coding region. It consists of five enhancer regions (E1–E5) with multiple binding sites for transcription factors. These transcription factors recruit transcriptional co-factors, in particular the BET bromodomain protein BRD4 and the SwItch/Sucrose non-fermentable (SWI/SNF) protein BRG1. These chromatin-associated factors mediate c-*Myc* transcription via long-range chromatin looping and selective interaction with the *c*-*Myc* promoter [5–7]. On the other hand, we recently demonstrated that STAT5 transcriptional activity is regulated by BET proteins, including BRD2 [31]. We found that BRD2 is recruited along STAT5 at the proximal promoter and transcription start site of the conventional STAT5 target gene *Cis*, both upon IL-3-induced STAT5 activation and in cells expressing and transformed by constitutively active STAT5. BRD2 association with the *Cis* gene is lost upon treatment with the BET inhibitor (+)-JQ1 (thereafter referred to as JQ1 [43]), resulting in transcriptional inhibition [31]. Interestingly, deacetylase inhibitors also repress STAT5-mediated transcription by displacing BRD2 and preventing recruitment of the transcriptional machinery [12, 31]. Our data therefore suggest that STAT5 recruits BRD2 which, in turn, supports transcriptional activation by assisting the assembly of the preinitiation complex [12, 31]. Given that STAT5-induced expression of *c*-*Myc* is also inhibited by JQ1 [30, 31, 44], and given the absence of STAT5 binding activity within *c*-*Myc* promoter and gene locus, we tested the hypothesis that STAT5 binds to the 3′ super-enhancer and recruits BRD2 proteins to regulate *c*-*Myc* gene expression.

## Results

### *c*-*Myc* expression is induced by STAT5 and repressed by inhibitors of deacetylases and of BET proteins in Ba/F3-derived cells

**Fig. 1:**
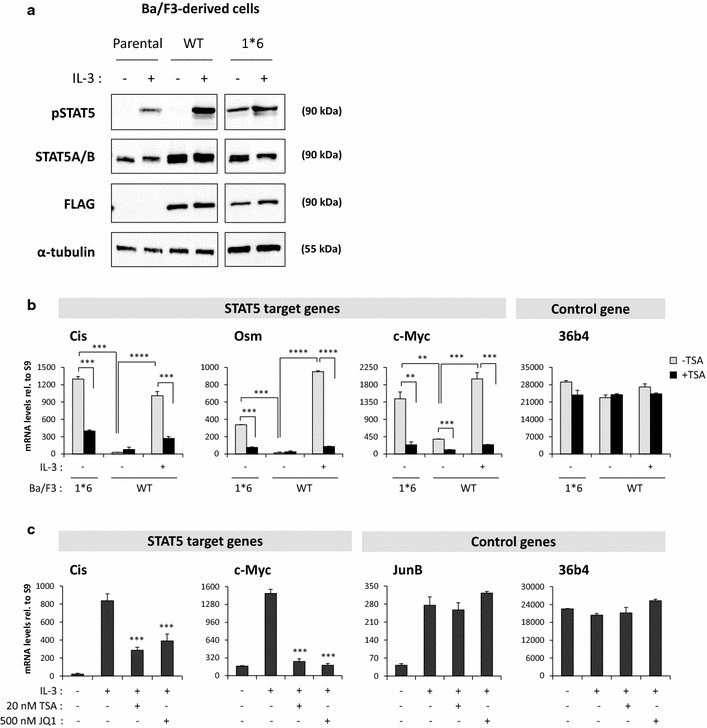
*c*-*Myc* gene expression in Ba/F3-derived cell lines. **a** STAT5 protein and phosphorylation levels in the parental Ba/F3 cells and in Ba/F3 cells stably expressing STAT5A-WT (Ba/F3-WT) and STAT5A-1*6 (Ba/F3-1*6). Ba/F3 and Ba/F3-WT cells, which grow in IL-3-containing medium, were withdrawn from IL-3 for 11 h and stimulated with IL-3 for 15 min. IL-3-independent Ba/F3-1*6 cells were stimulated with IL-3 in parallel. Brij whole-cell protein lysates (20 μg) were analysed by Western blot using antibodies specific for phosphorylated STAT5 (pSTAT5), total STAT5A and STAT5B (STAT5A/B), transgenic STAT5A-WT and STAT5A-1*6 (FLAG), and α-tubulin as a loading control. STAT5A/B signal in parental Ba/F3 cells corresponds to endogenous STAT5 protein levels, while the signals detected in the stable cell lines Ba/F3-WT and Ba/F3-1*6 represent both endogenous (STAT5A and STAT5B) and transgenic (STAT5A-WT or -1*6) proteins. **b** STAT5-mediated expression of *c*-*Myc* in Ba/F3-WT and -1*6 cells. Rested Ba/F3-WT cells stably expressing wild-type STAT5A were pre-treated 30 min with 200 nM TSA and further stimulated with IL-3 for 30 min. Ba/F3-1*6 cells expressing constitutively active STAT5A-1*6 were treated in parallel with 200 nM TSA for 60 min. Expression of STAT5 target genes (*Cis*, *Osm*, *c*-*Myc*) and of the housekeeping gene *36b4* was analysed by RT-qPCR. Expression of *c*-*Myc*, like that of *Cis* and *Osm*, was induced by STAT5A-WT and by constitutively active STAT5A-1*6, in an IL-3-dependent and -independent manner respectively. STAT5A-WT- and STAT5A-1*6-mediated expression of *c*-*Myc*, *Cis* and *Osm* was inhibited by the deacetylase inhibitor trichostatin A (TSA). Student’s t tests were employed to compare on the one hand IL-3-induced (WT) or 1*6-induced gene expression to the unstimulated WT control, and on the other hand TSA-treated to the vehicle control in each condition; **P < 0.01, ***P < 0.001, ****P < 0.0001; a *P* value <0.05 was considered statistically significant. **c** Expression of STAT5 target genes is inhibited by both deacetylase (TSA) and BET (JQ1) inhibitors. Ba/F3 cells were pre-treated with 20 nM TSA or 500 nM JQ1 for 30 min and stimulated with IL-3 for 60 min. Expression of STAT5 target genes (*Cis*, *c*-*Myc*) and of control genes (IL-3-dependent MAPK target gene *JunB* and housekeeping gene *36b4*) was analysed by RT-qPCR, as above. IL-3-induced expression of *Cis* and *c*-*Myc*, but not that of *JunB*, was inhibited by TSA and JQ1. One-way ANOVA with Dunnett’s multiple comparison test was used to assess differences between TSA- or JQ1-treated conditions and the vehicle-treated IL-3-stimulated control; ***P < 0.001; a *P* < 0.05 was considered statistically significant

This study was conducted in the mouse IL-3-dependent pro-B cell line Ba/F3 [45] and in its transformed IL-3-independent counterpart Ba/F3-1*6, which expresses the constitutively active mutant STAT5A-1*6 [35]. Whereas STAT5 phosphorylation, binding to DNA and transcriptional activity are induced by IL-3 in Ba/F3 and Ba/F3-WT cells, they are constitutive in Ba/F3-1*6 cells in the absence of IL-3 (Fig. 1a, b and [11, 12, 29, 31, 35]), hence mimicking the situation found in cancer cells. As such, Ba/F3 (or Ba/F3-WT) and Ba/F3-1*6 cells represent ideal experimental models to study and compare STAT5-mediated transcription in normal and cancer cells respectively. Similarly to that of the STAT5 target genes *Cis* and *Osm* [12, 31], expression of *c*-*Myc* was induced by IL-3 in cells expressing wild-type STAT5 while it was upregulated in cells expressing the STAT5A-1*6 mutant in the absence of IL-3 (Fig. 1b). In both cell lines, expression of *Cis*, *Osm* and *c*-*Myc* was inhibited by the deacetylase inhibitor trichostatin A (TSA) (Fig. 1b), in agreement with our previous reports that deacetylase inhibitors (notably TSA, valproic acid, apicidin, sodium butyrate and clinically relevant SAHA) prevent STAT5-mediated transcription [12, 31]. Likewise, STAT5-mediated expression of *Cis* and *c*-*Myc* in Ba/F3 cells was inhibited by the BET inhibitor JQ1 (Fig. 1c), as already reported [6, 30, 31, 44]. Expression of the IL-3-inducible but STAT5-independent gene *JunB* was not inhibited by TSA or JQ1 (Fig. 1c) while expression of the housekeeping gene *36b4* remained unaffected in all conditions (Fig. 1b, c).

### STAT5 binds to *c*-*Myc* super-enhancer

**Fig. 2:**
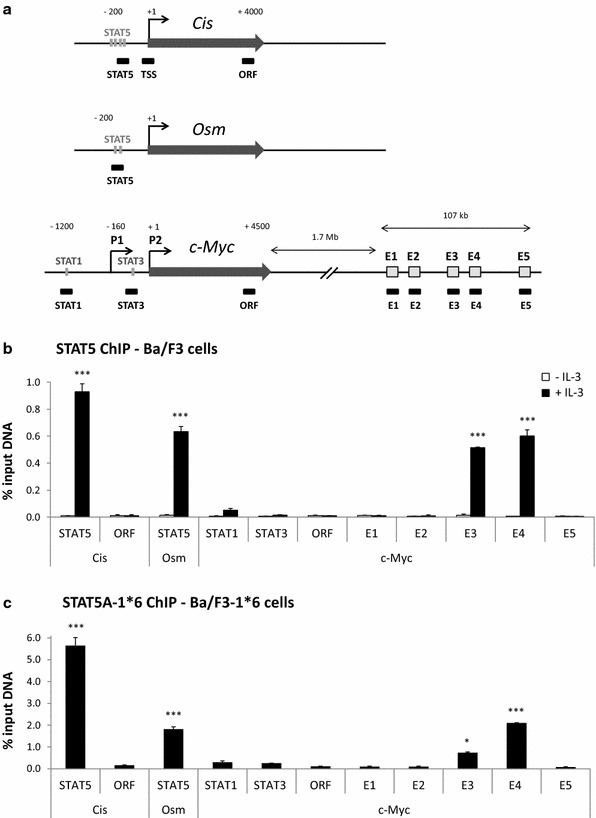
STAT5 specifically binds to the E3 and E4 regions of *c*-*Myc* super-enhancer. **a** Schematic representation of the mouse STAT5 target genes *Cis*, *Osm* and *c*-*Myc* and of the qPCR amplicons analysed following chromatin immunoprecipitation. Nucleotide positions are relative to the respective transcription start sites (TSS). Functional STAT binding sites within the individual promoter regions are indicated as *grey bars*. P1 and P2 designate *c*-*Myc* dual promoters, P2 being predominant in normal cells. E1 to E5 symbolise the five domains of *c*-*Myc* 3′ super-enhancer. *Black boxes* underneath the respective genes represent the qPCR amplicons. Primers amplifying regions within *Cis* and *c*-*Myc* open reading frame (ORF) were used as controls. **b** Chromatin immunoprecipitation (ChIP) was performed with whole-cell lysates from Ba/F3 cells (conventional ChIP protocol), either unstimulated or stimulated 30 min with IL-3, using antibodies specific for STAT5A + STAT5B (STAT5 ChIP). Immunoprecipitated genomic DNA (gDNA) was analysed by qPCR using primers shown in **a**. One-way ANOVA with Dunnett’s post test was used to evaluate IL-3-induced STAT5 enrichment at the various loci compared to the signal detected at the *c*-*Myc* “ORF” region, used as a reference and background control; ***P < 0.001; a *P* < 0.05 was considered statistically significant. **c** ChIP was conducted with nuclear lysates (alternative ChIP protocol) from Ba/F3-1*6 cells grown in the absence of IL-3, using antibodies specific for STAT5A, which recognize STAT5A-1*6 mutant. Immunoprecipitated gDNA was analysed by qPCR, as in **b**. One-way ANOVA with Dunnett’s multiple comparison test was used to evaluate STAT5 enrichment at the various loci vs. *c*-*Myc* “ORF” region, used as a reference and background control; *P < 0.05, ***P < 0.001

**Fig. 3:**
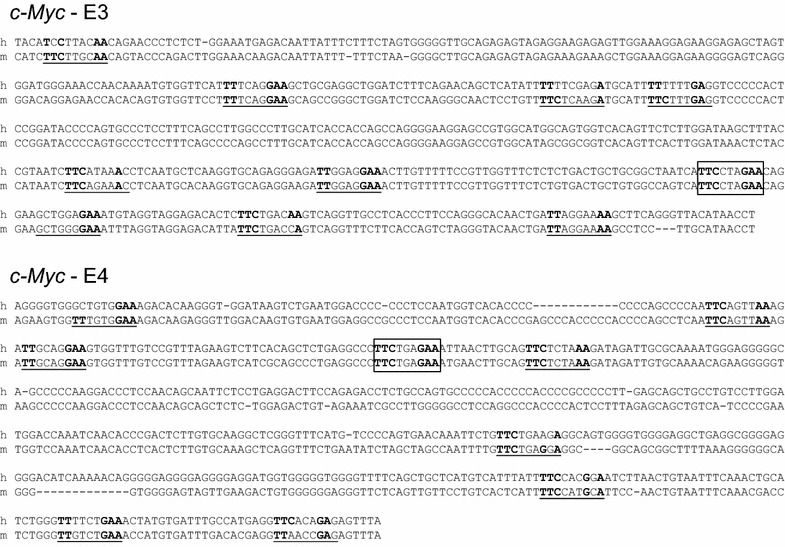
Sequence alignment of the mouse and human enhancer regions E3 and E4 located 3′ of the *c*-*Myc* gene. Consensus STAT5 binding sites (TTCNNNGAA) that are conserved between mouse and human sequences are designated as *white boxes*. Non-consensus STAT5 binding sites (usually TTCNNNNAA or TTNNNNGAA) are *underlined* [46]. E3 and E4 contain each one consensus and a cluster of 8–9 non-consensus putative STAT5 binding motifs that are well-conserved in both mouse and human genomes at the same position. Such pattern of conserved motifs was absent from E1, E2 and E5 elements (not shown)

Ba/F3 and Ba/F3-1*6 cells were used to investigate transcription factor recruitment along the *c*-*Myc* gene locus in normal and STAT5-transformed cells respectively. Chromatin immunoprecipitation (ChIP) assays were conducted in Ba/F3 and Ba/F3-1*6 cells using STAT5-specific antibodies. Immunoprecipitated genomic DNA was analysed by quantitative PCR using primers specific for the *c*-*Myc* gene locus—including known STAT binding sites—and for its 3′ super-enhancer (E1 to E5). Primers specific for regions encompassing the STAT5 binding sites of the *Cis* and *Osm* genes were investigated as controls (Fig. 2a). Upon IL-3 stimulation of Ba/F3 cells, STAT5 was specifically detected at the STAT5 binding sites present within the proximal promoter of the *Cis* and *Osm* genes (Fig. 2b). With the exception of a very weak STAT5 binding at a previously described STAT1 binding site within *c*-*Myc* promoter [40], no STAT5 binding was detected within the *c*-*Myc* locus. By contrast, a strong signal was detected specifically at the E3 and E4 regions of *c*-*Myc* super-enhancer (Fig. 2b). Interestingly, a similar binding pattern was found in Ba/F3-1*6 cells (Fig. 2c), demonstrating that both IL-3-induced wild-type STAT5 and constitutively activated STAT5-1*6 bind to *c*-*Myc* super-enhancer elements E3 and E4. In support of STAT5 binding to E3 and E4, sequence analysis of the *c*-*Myc* super-enhancer revealed the presence of clusters of putative STAT5 binding sites within E3 and E4, both consensus (TTCNNNGAA) and non-consensus [46], conserved between mouse and human genomes at the same position (Fig. 3). No conserved putative STAT5 binding sites were identified within E1, E2 and E5 (not shown).

### BRD2 co-localizes with constitutively active STAT5 at *c*-*Myc* super-enhancer

**Fig. 4:**
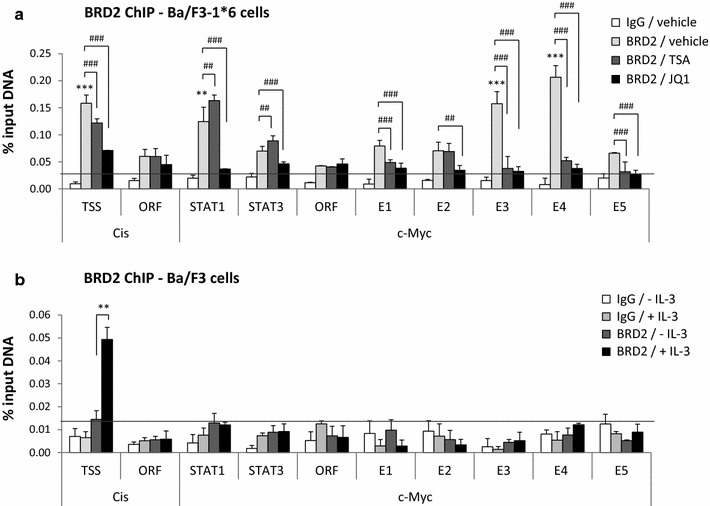
BRD2 co-localizes with STAT5 at E3/E4 in Ba/F3 cells transformed by constitutive active STAT5A-1*6. **a** Ba/F3-1*6 cells were treated for 60 min with 200 nM TSA, 1 μM JQ1 or 0.02 % DMSO (vehicle). Nuclei isolation and ChIP was performed following the alternative protocol, and using BRD2-specific antibodies or the same amount of rabbit IgG as a control. qPCR primers are depicted in Fig. 2a. Only the vehicle-treated IgG control is shown. TSA- and JQ1-treated IgG controls exhibited similar background levels (data not shown), as previously reported [31]. BRD2 binds strongly to the *Cis* transcription start site (TSS), the *c*-*Myc* promoter region (STAT1) and enhancer regions E3 and E4. BRD2 association is lost upon JQ1 treatment and is reduced at *Cis* TSS and *c*-*Myc* E3/E4 upon treatment with the deacetylase inhibitor TSA. Background cut-off (*horizontal line*) was defined as the mean of the signal generated by the IgG negative controls plus 2x the standard deviation (SD) of the IgG control (mean IgG + 2x SD). BRD2 enrichment at the various loci in the vehicle-treated condition was evaluated using One-way ANOVA with Dunnett’s multiple comparison vs. *c*-*Myc* “ORF” region, used as a reference; of note, BRD2 signal intensity at the *c*-*Myc* “ORF” region is not reduced upon JQ1 treatment, suggesting that it corresponds to background BRD2 signal; **P < 0.01, ***P < 0.001; a *P* < 0.05 was considered statistically significant. One-way ANOVA with Dunnett’s post test was used to assess differences between TSA- or JQ1-treated conditions and the vehicle-treated control; ^##^ *P* < 0.01, ^###^ *P* < 0.001; a *P* < 0.05 was considered statistically significant. **b** Ba/F3 cells were stimulated with IL-3 for 30 min and BRD2 ChIP was conducted with whole-cell lysates using BRD2-specific antibodies or rabbit IgG, following the conventional ChIP protocol [12, 31]. Genomic DNA was analysed as in **a**. Only background signals were detected along the *c*-*Myc* gene locus and downstream super-enhancer in Ba/F3 cells, while BRD2 was recruited in response to IL-3 at the transcription start site (TSS) of the *Cis* gene. Background cut-off (*horizontal line*) was defined as described in A (mean IgG + 2x SD). Student’s t test was used to monitor IL-3-induced BRD2 recruitment at *Cis* TSS; **P < 0.01; a *P* < 0.05 was considered statistically significant

Given that BRD4 is recruited at *c*-*Myc* super-enhancer in hematopoietic and leukemic cells [5, 6], and since we recently showed the implication of BRD2 in STAT5-mediated transcription in Ba/F3 and Ba/F3-1*6 cells [31], we investigated the recruitment of BRD2 along the *c*-*Myc* gene and super-enhancer by chromatin immunoprecipitation (Fig. 4). In cells expressing STAT5A-1*6, BRD2 bound at E3 and E4, but not at E1, E2 and E5 of *c*-*Myc* super-enhancer (Fig. 4a). Unexpectedly, BRD2 was also detected at the reported STAT1 binding site within the *c*-*Myc* promoter. As previously described [31], BRD2 was found proximate to the transcription start site of the *Cis* gene (Fig. 4a). BRD2 binding at *Cis* and *c*-*Myc* was lost upon treatment with the BET inhibitor JQ1 (Fig. 4a), demonstrating BRD2 binding specificity. Interestingly, BRD2 binding was reduced at *Cis* transcription start site and abolished at *c*-*Myc* E3/E4 upon TSA treatment, but not at the *c*-*Myc* promoter (STAT1) location. We previously showed that TSA-induced inhibition of STAT5 transcriptional activity correlates with BRD2 loss at the STAT5 target gene *Cis* [31]. We therefore show here that, similarly, BRD2 binding at E3/E4—but not at STAT1 element within its promoter—along with STAT5, strictly correlates with *c*-*Myc* transcriptional activation. Our data thus strongly suggest that BRD2 association with *c*-*Myc* super-enhancer is involved in STAT5-mediated transcription of *c*-*Myc*. The presence of BRD2 around the STAT1 binding site and its insensitivity to TSA, suggest that BRD2 is recruited in a STAT5-independent manner to the *c*-*Myc* promoter, possibly by other transcription factors [5–7], and that BRD2 association at this site is not implicated in STAT5-mediated transcription of the *c*-*Myc* gene.

To better characterize the role of STAT5 in BRD2 recruitment to STAT5 target genes, we next addressed whether STAT5A-1*6 and BRD2 physically interact. Co-immunoprecipitation experiments were carried out on nuclear lysates from formaldehyde-crosslinked Ba/F3-1*6 cells using STAT5A- and BRD2-specific antibodies and following the ChIP protocol (Additional file 1: Fig. S1A–C). Upon immunoprecipitation (IP), samples were analysed by Western blot (Additional file 1: Fig. S1B) and quantitative PCR as a control (Additional file 1: Fig. S1C). Recruitment efficiency and specificity of STAT5A-1*6 and BRD2 to the *Cis* gene was comparable to that observed before (Additional file 1: Fig. S1C). No co-immunoprecipitation of STAT5A-1*6 and BRD2 was detected in Western blot, following neither STAT5A nor BRD2 IP (Additional file 1: Fig. S1B). It should be noted that, while STAT5A was strongly immunoprecipitated under the ChIP experimental conditions, BRD2 was poorly pulled-down in the same conditions (Additional file 1: Fig. S1B), probably explaining the weak signals usually detected by qPCR following BRD2 ChIP (Additional file 1: Fig. S1C; Fig. 4a), but also making it difficult to evidence a co-immunoprecipitation with STAT5A. The weak immunoprecipitation efficiency observed in BRD2 ChIP is likely the consequence of the experimental conditions used (formaldehyde-mediated crosslinking and/or IP buffer composition). Indeed, conventional immunoprecipitation from nuclear lysates of non-crosslinked cells (Additional file 1: Fig. S1D) and using mild buffer conditions resulted in efficient BRD2 immunoprecipitation (Additional file 1: Fig. S1E). However, no co-immunoprecipitation of STAT5A was observed in these conditions. Altogether, these experiments suggest that STAT5A-1*6 and BRD2 do not directly interact but are rather co-recruited at the chromatin level.

Finally, we investigated BRD2 association with the *c*-*Myc* gene and its super-enhancer in IL-3-stimulated Ba/F3 cells. While BRD2 was recruited upon IL-3 stimulation at the transcription start site of the *Cis* gene, as previously described [31], no BRD2 enrichment above the IgG background was detected along the *c*-*Myc* gene and downstream enhancer in the same conditions (Fig. 4b). This observation suggests that, by contrast to STAT5A-1*6, IL-3-induced wild-type STAT5 might not be efficient in recruiting and/or stabilizing BRD2 at *c*-*Myc* enhancer.

## Discussion

This study identified functional STAT5 binding sites possibly regulating *c*-*Myc* transcription. We showed that STAT5 specifically binds to enhancer regions E3 and E4 of *c*-*Myc* 3′ super-enhancer. Putative STAT5 binding sites within E3/E4 are organised in clusters of consensus and non-consensus binding motifs. Similar functional STAT5 binding sites have been described at other STAT5 target genes [13]. Binding of wild-type STAT5 to E3/E4 was induced by IL-3 in normal Ba/F3 cells while STAT5A-1*6 binding was constitutive and independent of IL-3 in transformed Ba/F3-1*6 cells. Interestingly, ChIP-Seq assays performed in human HEL leukemia cells revealed that JAK2^V617F^-induced constitutive active STAT5 [47, 48] is bound at *c*-*Myc* enhancer region E3 (ArrayExpress accession number E-MTAB-1096; [49]), providing another evidence of STAT5 constitutive binding at *c*-*Myc* super-enhancer in leukemia cells.

Constitutive binding of STAT5 at E3/E4 in Ba/F3-1*6 cells coincided with that of BRD2, suggesting that STAT5 might play a role in BRD2 maintenance at *c*-*Myc* super-enhancer in transformed cells. By contrast, BRD2 was not detected by ChIP at the *c*-*Myc* locus in IL-3-stimulated Ba/F3 cells. We cannot exclude at this point that this absence of specific signal is due to a detection problem. Alternatively, it might be the consequence of transient STAT5 binding in Ba/F3 cells, as opposed to constitutive STAT5 binding in Ba/F3-1*6 cells which might be necessary for BRD2 maintenance at *c*-*Myc* super-enhancer. We could not evidence an interaction between STAT5A-1*6 and BRD2 in co-immunoprecipitation assays. Therefore, our data suggest that constitutive binding of STAT5A-1*6 at E3/E4 might—directly or indirectly—assist and/or stabilize BRD2 association with *c*-*Myc* super-enhancer. Furthermore, it remains possible that other BET protein(s) are involved in the regulation of *c*-*Myc* by wild-type STAT5 in Ba/F3 cells. This proposition is supported by our previous observation that BRD2 was detected at the *Cis* but not at the *Osm* promoter in IL-3-stimulated Ba/F3 cells [31]. This is also in agreement with the implication of BRD4 in long-range regulation of *c*-*Myc* transcription via its 3′ super-enhancer [5, 6]. Interestingly, it was shown that multiple transcription factors, the BET protein BRD4 and the SWI/SNF component BRG1 are recruited to *c*-*Myc* 3′ enhancer and contribute to *c*-*Myc* gene transcription [5, 6]. Notably, BRG1 was proposed to maintain transcription factor occupancy at the enhancer region and to facilitate interactions with the *c*-*Myc* promoter [5]. Whether such a mechanism of stabilisation by BRG1 also takes place in STAT5-mediated transcription remains to be shown. Maintenance of STAT5 occupancy at *c*-*Myc* super-enhancer would be an attractive explanation for the absence of effect of STAT5 (partial) knock-down on *c*-*Myc* gene expression [13].

## Conclusions

We showed that constitutive binding of STAT5 and maintenance of BRD2 at E3/E4 in transformed Ba/F3-1*6 cells correlated with transcriptional activation of *c*-*Myc*. Furthermore, BRD2 binding at E3/E4 was lost upon TSA- and JQ1-mediated inhibition of *c*-*Myc* expression, both of which inhibit STAT5-induced transcription [31]. Our data therefore suggest that constitutive binding of STAT5A-1*6 contributes to BRD2 maintenance at *c*-*Myc* super-enhancer in transformed Ba/F3-1*6 cells, which in turn might be implicated in *c*-*Myc* overexpression. In support of a role of STAT5 in *c*-*Myc* overexpression in leukemia via *c*-*Myc* super-enhancer, E3 was recently shown to display enhancer activity in K562 leukemia cells, using a luciferase reporter assay [5]. This finding nicely fits with our previous finding that *c*-*Myc* expression in K562 cells is dependent on BCR-ABL-induced constitutive active STAT5 [32]. Our model is hence in line with the recently suggested leukemia maintenance function attributed to *c*-*Myc* 3′ super-enhancer, via the recruitment of BET proteins by hematopoietic transcription factors [5, 6, 8]. We propose that the transcription factor STAT5 might play a similar role in *c*-*Myc* overexpression, in leukemia exhibiting constitutive STAT5 activation. Further functional assays will be necessary to verify this proposition.

## Methods

### Chemicals

Dimethyl sulfoxide (DMSO) and trichostatin A (TSA) were purchased from SIGMA (D-2650 and T-8552 respectively). (+)-JQ1 (BPS Bioscience #27401)—hereafter abbreviated to JQ1—was purchased from BIOMOL GmbH. TSA and JQ1 were dissolved in DMSO at a final concentration of 1 mM (TSA) and 5 mM (JQ1). DMSO was used as vehicle control. Its final concentration was adjusted to 0.02 % in all conditions.

### Cells

All cell lines were cultivated at 37 °C under 5 % CO_2_ in a humidified incubator. The interleukin-3 (IL-3)-dependent mouse pro-B cell line Ba/F3 (a kind gift from Jacqueline Marvel, IFR 128 BioSciences Gerland-Lyon Sud, France [45]) was grown in RPMI 1640 (PAN-Biotech P04-16500) supplemented with 10 % heat-inactivated fetal calf serum (FCS; PAN-Biotech), penicillin/streptomycin (100 U/mL penicillin, 100 μg/mL streptomycin; PAN-Biotech) and 2 ng/ml rmIL-3 (ImmunoTools). No ethical approval was required to obtain and use the Ba/F3 parental cell line. Ba/F3-derived cell lines were generated according to German GenTSV (genetic engineering safety regulations; authorization AZ.55.1-8791.7.52). The IL-3-independent Ba/F3-1*6 cell line (clone F7) stably expressing the FLAG-tagged constitutively active mouse STAT5A-1*6 mutant [35] has been described [32], and was grown in RPMI 1640 supplemented with 10 % heat-inactivated FCS, penicillin/streptomycin and 600 μg/ml G418 (SIGMA A-1720). The IL-3-dependent Ba/F3-WT cell line (clone A7) expressing FLAG-tagged wild-type (WT) mouse STAT5A was generated by electroporating Ba/F3 cells with a pcDNA3-based expression vector allowing expression of a mSTAT5A-WT-FLAG fusion protein. Stably transfected cells were selected in IL-3-containing medium in the presence of 800 μg/mL G418 (PAA). Individual clones were isolated and characterized to verify mSTAT5A-WT transgene expression and proper IL-3-dependent activation of STAT5A-WT. Ba/F3-WT clone A7 was used for this study and served as a control for potential adverse effects of FLAG-tagged protein overexpression. Ba/F3-WT cells were maintained in RPMI 1640 supplemented with 10 % heat-inactivated FCS, penicillin/streptomycin, 600 μg/ml G418 (SIGMA A-1720) and 2 ng/ml rmIL-3. For cytokine stimulation of Ba/F3 and Ba/F3-WT cells, cells were washed twice in RPMI 1640 and rested in RPMI 1640, 10 % FCS, penicillin/streptomycin for 6–12 h before addition of 5 ng/ml IL-3 for 15–120 min, as indicated. For inhibitor treatment of Ba/F3 and Ba/F3-WT cells, rested cells were pre-treated 30 min with the respective compound or with DMSO (vehicle) prior to IL-3 stimulation. Ba/F3-1*6 cells were treated with inhibitors or vehicle for 60 min.

For co-immunoprecipitation assays from non-crosslinked cells, previously described Ba/F3-tet-on-1*6 cells conditionally expressing STAT5A-1*6 in the presence of doxycycline were used [31]. Briefly, Ba/F3-tet-on-1*6 cells were grown for 9 h in the presence of 1 μg/ml doxycycline in IL-3-free medium. Cells were harvested for nuclear fractionation and western blot analysis.

### Gene expression analysis by RT-qPCR

Following inhibitor and cytokine treatments, cells were harvested, cDNA synthesized and quantitative PCR performed as previously described [31]. Nucleotide sequence of the qPCR primers used in this study have been published [12, 13, 31]. Data were normalized to mouse S9 ribosomal mRNA and expressed as relative mRNA levels, like previously reported [12, 31]. Data are mean ± SD of the quantitative PCR performed in either duplicate or triplicate, and are representative of at least two independent experiments.

### Chromatin immunoprecipitation (ChIP)

Chromatin immunoprecipitation (ChIP) was carried out from either whole-cell (conventional protocol) or nuclear (alternative protocol) lysates, following reported procedures [12, 31, 50]. Chromatin Immunoprecipitation from nuclear lysates yields stronger signals than from whole cell lysates [50]. Antibodies used were as follows: STAT5A (Santa Cruz Biotechnology sc-1081), STAT5A + B (Santa Cruz Biotechnology sc-835), BRD2 (Bethyl A302-583A) and IgG from rabbit serum (SIGMA I-5006). Antibody concentrations used were as reported [12, 31]. Co-precipitated genomic DNA was measured by quantitative PCR. Mouse *Cis*- and *Osm*-specific primers have been described [31]. Mouse *c*-*Myc*-specific forward and reverse primers were the following, respectively: STAT1, TTTATTCTAGGGTCTCTGCAGGC and GAAAACCCGGACTTCCCAG; STAT3, CCCTCCTGCCTCCTGAAGG and CAGGATCCCTCCCCTCCC; ORF, AACAACCGCAAGTGCTCCAG and GTCGTTTTCCTCCGTGTCTGAG; E1, ACGCTCAGAGTGCTTTCCAT and GGTGGTGTGGGGTGACTAATAT; E2, GTGGGAGGGACTGAAATGGAG and TGGGCAAAGCTAGAGGCAGAT; E3, GAACAGGAAGCTGGGGAAAT and TGCAAGGAGGCTTTTCCTAA; E4, CACCCCAGCCTCAATTCAGT and GCTGCGATGACTTCTAAACGG; E5, GCAACAGCAAGAACCAGTGA and TGCTTCTCCTGAACCACCTT. Results are expressed as percentage (%) of input DNA. Data are mean ± SD of the quantitative PCR performed in either duplicate or triplicate, and are representative of at least two independent experiments.

### Protein analysis by immunoprecipitation and Western blot

Western blots were performed as described [32], using the following antibodies and respective dilutions: pSTAT5 (#9351, Cell Signaling Technology; 1:1000), STAT5A (L-20, sc-1081, Santa-Cruz Biotechnology; 1:1000), STAT5A/B (C-17, sc-835, Santa-Cruz Biotechnology; 1:1000), FLAG (M2, SIGMA F-1804; 1:500), Brd2 (Bethyl A302–583A; 1:2000), α-tubulin (DM1A, sc-32293, Santa-Cruz Biotechnology; 1:200), HDAC1 (Millipore 05-100; 1:1000), Anti-Rabbit IgG-Peroxidase (SIGMA A0545; 1:10,000), Anti-Mouse IgG-Peroxidase (SIGMA A8924; 1:10,000). HDAC1 and α-tubulin were used as nuclear and cytosolic protein markers respectively to control the quality of the nuclear fractionation, like previously reported [31].

Brij whole-cell protein lysis and nuclear fractionation from non-crosslinked cells were conducted as previously described [31]. Immunoprecipitations from non-crosslinked Ba/F3-tet-on-1*6 cells was performed using 250 μg nuclear protein lysate diluted 1:10 in Brij buffer (10 mM Tris–HCl pH 7.5, 150 mM NaCl, 2 mM EDTA pH 8.0, 0.875 % Brij 97, 0.125 % NP40, 10 mM NaF, 1 mM Na_3_VO_4_, 10 μg*/*ml leupeptin, 10 μg*/*ml aprotinin, 0.5 mM phenylmethylsulfonyl fluoride) and 1.2 μg BRD2 antibody (Bethyl A302–583A) or 1.2 μg rabbit IgG (SIGMA I-5006) as a negative control. Immunoprecipitations were conducted for 5 h and immunocomplexes were collected using protein A-Sepharose beads. Consecutive to washing in Brij buffer, beads were boiled in 60 μl Laemmli buffer. Half of the eluted bead fraction was analyzed by Western blot in parallel to input lysate (3 % or 8 μg nuclear proteins) and immunoprecipitation supernatants (same volume as input lysate).

Nuclear-enriched protein lysate preparation and immunoprecipitations (IP) from formaldehyde-crosslinked Ba/F3-1*6 cells were carried out following the alternative ChIP protocol described above [31, 50], using 7 × 10^6^ cells for the nuclei preparation and 2.4 μg antibody (rabbit IgG, STAT5A or BRD2) per IP. Subsequent to the last washing step, ~20 % beads were further processed for genomic DNA isolation and qPCR analysis, following the ChIP protocol. The remaining beads were boiled 10 min in 50 μl Laemmli buffer and ~40 % of the eluted bead fraction (20 μl) was analysed by Western blot in parallel to input lysate (3 %) and immunoprecipitation supernatants (same volume as input lysate).

## Additional file

10.1186/s12867-016-0063-y Protein interaction between STAT5A-1*6 and BRD2 cannot be evidenced in co-immunoprecipitation assays. Nuclear lysates from formaldehyde-crosslinked (A-C) or non-crosslinked (D, E) STAT5A-1*6-expressing cells were prepared as described in the Methods section. Nuclear protein enrichment was verified by Western blot using antibodies specific for the nuclear and cytosolic proteins HDAC1 and α-tubulin respectively, and STAT5A-1*6 expression was monitored using the FLAG antibody (A, D). Immunoprecipitations (IP) were performed as described in the “Methods” section using the indicated antibodies. Input (In), immunoprecipitation supernatants (SN) and eluted bead fractions (B) were analysed by immunoblot (IB) using the indicated antibodies (B, E). In panel B, arrow points to BRD2 and (*) indicates a non-specific signal associated with the bead fractions. Bead samples from the IP experiment shown in panel B (crosslinked cells) were further processed for ChIP analysis by qPCR, using the *Cis*-specific primers depicted in Fig. 2a (C). In panel C, background cut-off (dotted line) was defined as in legend to Fig. 4 (mean IgG background + 2x SD). One-way ANOVA with Dunnett’s multiple comparison test was used to evaluate BRD2 and STAT5 enrichment at the STAT5 binding site (STAT5) and transcription start site (TSS) of the *Cis* gene, in comparison to the “ORF” region, used as a reference and background control; ****P* < 0.001; a *P* value < 0.05 was considered statistically significant.

## Abbreviations

Ba/F3: mouse pro-B IL-3-dependent cell line
Ba/F3-1*6: IL-3-independent BaF3 cell line stably expressing constitutive active STAT5A-1*6
BCR-ABL: tyrosine kinase oncogenic fusion
BET: bromodomain and extra-terminal protein
BRD2 and BRD4: bromodomain-containing protein 2 and 4 respectively, members of the BET protein family
BRG1: Brahma-related gene-1 encoding an ATPase and component of the SWI/SNF complex
DMSO: dimethyl sulfoxide
GAS element: Gamma interferon activation site (STAT-responsive) element
IL-2 and IL-3: interleukin-2 and -3
(+)-JQ1: inhibitor of BET protein function
K562: human leukemic cell line
SAHA: suberoylanilide hydroxamic acid
STAT5: signal transducer and activator of transcription 5
STAT5A-1*6: constitutively active STAT5A mutant
SWI/SNF: SWItch/Sucrose Non-Fermentable, chromatin remodelling complex
TSA: trichostatin A

## References

[1] Hoffman B, Amanullah A, Shafarenko M, Liebermann DA (2002). The proto-oncogene c-myc in hematopoietic development and leukemogenesis. Oncogene.

[2] Chappell J, Dalton S (2013). Roles for MYC in the establishment and maintenance of pluripotency. Cold Spring Harb Perspect Med.

[3] McKeown MR, Bradner JE (2014). Therapeutic strategies to inhibit MYC. Cold Spring Harb Perspect Med.

[4] Gabay M, Li Y, Felsher DW (2014). MYC activation is a hallmark of cancer initiation and maintenance. Cold Spring Harb Perspect Med.

[5] Shi J, Whyte WA, Zepeda-Mendoza CJ, Milazzo JP, Shen C, Roe J-S (2013). Role of SWI/SNF in acute leukemia maintenance and enhancer-mediated Myc regulation. Genes Dev.

[6] Roe J-S, Mercan F, Rivera K, Pappin DJ, Vakoc CR (2015). BET bromodomain inhibition suppresses the function of hematopoietic transcription factors in acute myeloid leukemia. Mol Cell.

[7] Sotelo J, Esposito D, Duhagon MA, Banfield K, Mehalko J, Liao H (2010). Long-range enhancers on 8q24 regulate c-Myc. Proc Natl Acad Sci USA.

[8] Yashiro-Ohtani Y, Wang H, Zang C, Arnett KL, Bailis W, Ho Y (2014). Long-range enhancer activity determines Myc sensitivity to Notch inhibitors in T cell leukemia. Proc Natl Acad Sci USA.

[9] Sengupta D, Kannan A, Kern M, Moreno MA, Vural E, Stack B (2015). Disruption of BRD4 at H3K27Ac-enriched enhancer region correlates with decreased c-Myc expression in Merkel cell carcinoma. Epigenetics.

[10] Lord JD, McIntosh BC, Greenberg PD, Nelson BH (1950). The IL-2 receptor promotes lymphocyte proliferation and induction of the c-myc, bcl-2, and bcl-x genes through the trans-activation domain of Stat5. J Immunol Baltim Md.

[11] Nosaka T, Kawashima T, Misawa K, Ikuta K, Mui AL, Kitamura T (1999). STAT5 as a molecular regulator of proliferation, differentiation and apoptosis in hematopoietic cells. EMBO J.

[12] Rascle A, Johnston JA, Amati B (2003). Deacetylase activity is required for recruitment of the basal transcription machinery and transactivation by STAT5. Mol Cell Biol.

[13] Basham B, Sathe M, Grein J, McClanahan T, D’Andrea A, Lees E (2008). In vivo identification of novel STAT5 target genes. Nucleic Acids Res.

[14] Wakao H, Gouilleux F, Groner B (1994). Mammary gland factor (MGF) is a novel member of the cytokine regulated transcription factor gene family and confers the prolactin response. EMBO J.

[15] Grimley PM, Dong F, Rui H (1999). Stat5a and Stat5b: fraternal twins of signal transduction and transcriptional activation. Cytokine Growth Factor Rev.

[16] Nosaka T, Kitamura T (2002). Pim-1 expression is sufficient to induce cytokine independence in murine hematopoietic cells, but is dispensable for BCR-ABL-mediated transformation. Exp Hematol.

[17] Liu CB, Itoh T, Arai K, Watanabe S (1999). Constitutive activation of JAK2 confers murine interleukin-3-independent survival and proliferation of BA/F3 cells. J Biol Chem.

[18] Gesbert F, Griffin JD (2000). Bcr/Abl activates transcription of the Bcl-X gene through STAT5. Blood.

[19] Valentino L, Pierre J (2006). JAK/STAT signal transduction: regulators and implication in hematological malignancies. Biochem Pharmacol.

[20] Ren S, Cai HR, Li M, Furth PA (2002). Loss of Stat5a delays mammary cancer progression in a mouse model. Oncogene.

[21] Romanski A, Schwarz K, Keller M, Wietbrauk S, Vogel A, Roos J (2012). Deacetylase inhibitors modulate proliferation and self-renewal properties of leukemic stem and progenitor cells. Cell Cycle Georget Tex.

[22] Kosan C, Ginter T, Heinzel T, Krämer OH (2013). STAT5 acetylation: mechanisms and consequences for immunological control and leukemogenesis. JAK-STAT.

[23] Pietschmann K, Bolck HA, Buchwald M, Spielberg S, Polzer H, Spiekermann K (2012). Breakdown of the FLT3-ITD/STAT5 axis and synergistic apoptosis induction by the histone deacetylase inhibitor panobinostat and FLT3-specific inhibitors. Mol Cancer Ther.

[24] Evrot E, Ebel N, Romanet V, Roelli C, Andraos R, Qian Z (2013). JAK1/2 and Pan-deacetylase inhibitor combination therapy yields improved efficacy in preclinical mouse models of JAK2V617F-driven disease. Clin Cancer Res.

[25] Wang Y, Fiskus W, Chong DG, Buckley KM, Natarajan K, Rao R (2009). Cotreatment with panobinostat and JAK2 inhibitor TG101209 attenuates JAK2V617F levels and signaling and exerts synergistic cytotoxic effects against human myeloproliferative neoplastic cells. Blood.

[26] Nguyen T, Dai Y, Attkisson E, Kramer L, Jordan N, Nguyen N (2011). HDAC inhibitors potentiate the activity of the BCR/ABL kinase inhibitor KW-2449 in imatinib-sensitive or -resistant BCR/ABL+ leukemia cells in vitro and in vivo. Clin Cancer Res.

[27] Fiskus W, Sharma S, Qi J, Shah B, Devaraj SGT, Leveque C (2014). BET protein antagonist JQ1 is synergistically lethal with FLT3 tyrosine kinase inhibitor (TKI) and overcomes resistance to FLT3-TKI in AML cells expressing FLT-ITD. Mol Cancer Ther.

[28] Fiskus W, Sharma S, Qi J, Valenta JA, Schaub LJ, Shah B (2014). Highly active combination of BRD4 antagonist and histone deacetylase inhibitor against human acute myelogenous leukemia cells. Mol Cancer Ther.

[29] Rascle A, Lees E (2003). Chromatin acetylation and remodeling at the Cis promoter during STAT5-induced transcription. Nucleic Acids Res.

[30] Liu S, Walker SR, Nelson EA, Cerulli R, Xiang M, Toniolo PA (2014). Targeting STAT5 in hematological malignancies through inhibition of the bromodomain and extra-terminal (BET) bromodomain protein BRD2. Mol Cancer Ther.

[31] Pinz S, Unser S, Buob D, Fischer P, Jobst B, Rascle A (2015). Deacetylase inhibitors repress STAT5-mediated transcription by interfering with bromodomain and extra-terminal (BET) protein function. Nucleic Acids Res.

[32] Pinz S, Unser S, Brueggemann S, Besl E, Al-Rifai N, Petkes H (2014). The synthetic α-bromo-2′,3,4,4′-tetramethoxychalcone (α-Br-TMC) inhibits the JAK/STAT signaling pathway. PLoS One.

[33] de Groot RP, Raaijmakers JA, Lammers JW, Jove R, Koenderman L (1999). STAT5 activation by BCR-Abl contributes to transformation of K562 leukemia cells. Blood.

[34] Nieborowska-Skorska M, Wasik MA, Slupianek A, Salomoni P, Kitamura T, Calabretta B (1999). Signal transducer and activator of transcription (STAT)5 activation by BCR/ABL is dependent on intact Src homology (SH)3 and SH2 domains of BCR/ABL and is required for leukemogenesis. J Exp Med.

[35] Onishi M, Nosaka T, Misawa K, Mui AL, Gorman D, McMahon M (1998). Identification and characterization of a constitutively active STAT5 mutant that promotes cell proliferation. Mol Cell Biol.

[36] Silva M, Benito A, Sanz C, Prosper F, Ekhterae D, Nuñez G (1999). Erythropoietin can induce the expression of bcl-x(L) through Stat5 in erythropoietin-dependent progenitor cell lines. J Biol Chem.

[37] Xu M, Nie L, Kim S-H, Sun X-H (2003). STAT5-induced Id-1 transcription involves recruitment of HDAC1 and deacetylation of C/EBPbeta. EMBO J.

[38] Nelson EA, Walker SR, Alvarez JV, Frank DA (2004). Isolation of unique STAT5 targets by chromatin immunoprecipitation-based gene identification. J Biol Chem.

[39] Kiuchi N, Nakajima K, Ichiba M, Fukada T, Narimatsu M, Mizuno K (1999). STAT3 is required for the gp130-mediated full activation of the c-myc gene. J Exp Med.

[40] Ramana CV, Grammatikakis N, Chernov M, Nguyen H, Goh KC, Williams BR (2000). Regulation of c-myc expression by IFN-gamma through Stat1-dependent and -independent pathways. EMBO J.

[41] Grigorieva I, Grigoriev VG, Rowney MK, Hoover RG (2000). Regulation of c-myc transcription by interleukin-2 (IL-2). Identification of a novel IL-2 response element interacting with STAT-4. J Biol Chem.

[42] Uslu VV, Petretich M, Ruf S, Langenfeld K, Fonseca NA, Marioni JC (2014). Long-range enhancers regulating Myc expression are required for normal facial morphogenesis. Nat Genet.

[43] Filippakopoulos P, Qi J, Picaud S, Shen Y, Smith WB, Fedorov O (2010). Selective inhibition of BET bromodomains. Nature.

[44] Mertz JA, Conery AR, Bryant BM, Sandy P, Balasubramanian S, Mele DA (2011). Targeting MYC dependence in cancer by inhibiting BET bromodomains. Proc Natl Acad Sci USA.

[45] Palacios R, Steinmetz M (1985). Il-3-dependent mouse clones that express B-220 surface antigen, contain Ig genes in germ-line configuration, and generate B lymphocytes in vivo. Cell.

[46] Soldaini E, John S, Moro S, Bollenbacher J, Schindler U, Leonard WJ (2000). DNA binding site selection of dimeric and tetrameric Stat5 proteins reveals a large repertoire of divergent tetrameric Stat5a binding sites. Mol Cell Biol.

[47] Quentmeier H, MacLeod RA, Zaborski M, Drexler HG (2006). JAK2 V617F tyrosine kinase mutation in cell lines derived from myeloproliferative disorders. Leukemia.

[48] Bar-Natan M, Nelson EA, Walker SR, Kuang Y, Distel RJ, Frank DA (2012). Dual inhibition of Jak2 and STAT5 enhances killing of myeloproliferative neoplasia cells. Leukemia.

[49] Dawson MA, Foster SD, Bannister AJ, Robson SC, Hannah R, Wang X (2012). Three distinct patterns of histone H3Y41 phosphorylation mark active genes. Cell Rep.

[50] Pinz S, Rascle A. Assessing HDAC function in the regulation of signal transducer and activator of transcription 5 (STAT5) activity using chromatin immunoprecipitation (ChIP). Methods Mol Biol. 2016 **(in press)**. 10.1007/978-1-4939-6527-4_19 27761827

