# Supplementary material for: Signal transducer and activator of transcription STAT5 is recruited to c-Myc super-enhancer
Source: BMC Mol Biol. 2016 Apr 14;17:10. doi: 10.1186/s12867-016-0063-y (PMC4831086; doi:10.1186/s12867-016-0063-y)
Supplement: Supplementary file 1 — 10.1186/s12867-016-0063-y Protein interaction between STAT5A-1*6 and BRD2 cannot be evidenced in co-immunoprecipitation assays. Nuclear lysates from formaldehyde-crosslinked (A-C) or non-crosslinked (D, E) STAT5A-1*6-expressing cells were prepared as described in the Methods section. Nuclear protein enrichment was verified by Western blot using antibodies specific for the nuclear and cytosolic proteins HDAC1 and α-tubulin respectively, and STAT5A-1*6 expression was monitored using the FLAG antibody (A, D). Immunoprecipitations (IP) were performed as described in the “Methods” section using the indicated antibodies. Input (In), immunoprecipitation supernatants (SN) and eluted bead fractions (B) were analysed by immunoblot (IB) using the indicated antibodies (B, E). In panel B, arrow points to BRD2 and (*) indicates a non-specific signal associated with the bead fractions. Bead samples from the IP experiment shown in panel B (crosslinked cells) were further processed for ChIP analysis by qPCR, using the Cis-specific primers depicted in Fig. 2a (C). In panel C, background cut-off (dotted line) was defined as in legend to Fig. 4 (mean IgG background + 2x SD). One-way ANOVA with Dunnett’s multiple comparison test was used to evaluate BRD2 and STAT5 enrichment at the STAT5 binding site (STAT5) and transcription start site (TSS) of the Cis gene, in comparison to the “ORF” region, used as a reference and background control; ***P < 0.001; a P value < 0.05 was considered statistically significant. [file 12867_2016_63_MOESM1_ESM.pdf]

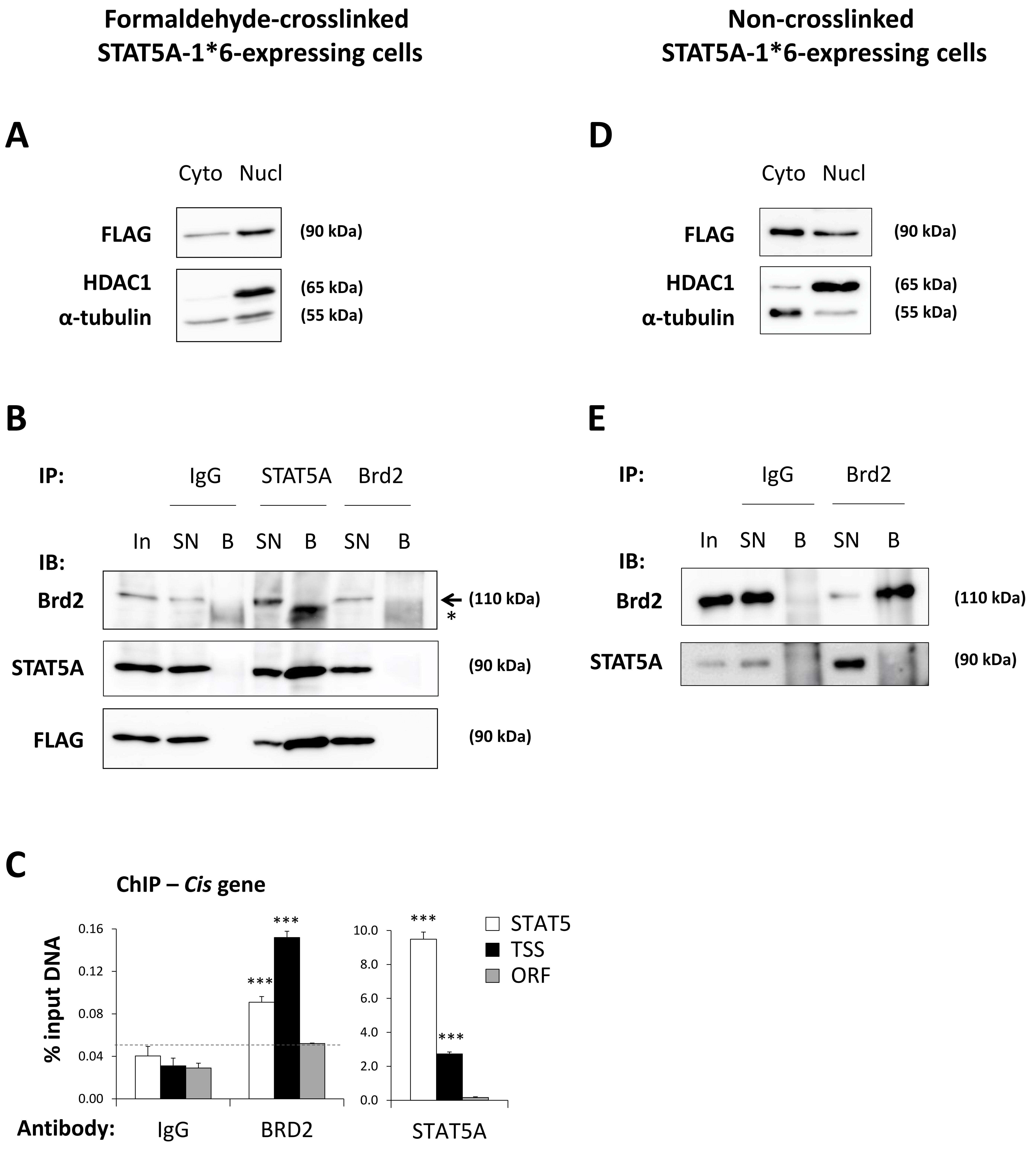

Figure S1 (Pinz et al.)
